# Thrips (Thysanoptera: Terebrantia) in Nectarine Orchards in North-East Spain: Species Diversity and Fruit Damage

**DOI:** 10.3390/insects15090699

**Published:** 2024-09-14

**Authors:** Albert Gallardo-Ferrand, Lucía Adriana Escudero-Colomar, Jesús Avilla, Dolors Bosch-Serra

**Affiliations:** 1 Institute of Agrifood Research and Technology (IRTA) Agronòms Lleida, Sustainable Vegetal Protection, Av. Alcalde Rovira Roure 191, 25198 Lleida, Spain; albert.gallardo@irta.cat; 2 Institute of Agrifood Research and Technology (IRTA) Mas Badia, Sustainable Vegeteal Protection, 17134 Girona, Spain; adriana.escudero@irta.cat; 3 Department of Agricultural and Forest Sciences and Engineering, Agrotecnio Center—CERCA, University of Lleida (UdL), Av. Alcalde Rovira Roure 191, 25198 Lleida, Spain

**Keywords:** nectarine, *Thrips fuscipennis*, *Frankliniella occidentalis*, silvering, organic orchards, integrated pest management, tree canopy

## Abstract

**Simple Summary:**

Thrips constitute one of the main nectarine pests, with damage either in flowering or before harvesting (silvering). Nectarine orchards under organic and integrated management were sampled in Lleida (Baix Segre intensive production area) and Girona at four key moments of the season (bud burst, full flowering, fruit setting stage, and colorization of the fruit) during 2021 and 2022 to determine the species composition and damage during fruit maturation. Fifteen species were collected in Lleida, and 10 species were collected in Girona. Organic orchards in Lleida showed lower populations and silvering damage levels than integrated orchards. *Thrips fuscipennis* Haliday (Thysanoptera: Thripidae) 1836 was the main species in Lleida during harvest, and *Frankliniella occidentalis* (Pergande) 1895 was the main species in Girona. Due to their predominance, both species were associated with silvering damage during fruit maturation.

**Abstract:**

Thrips constitute one of the main nectarine pests, with damage either in flowering or before harvesting (silvering). Several species are associated with damage to flowers, but *Frankliniella occidentalis* (Pergande) (Thysanoptera: Thripidae) is the main species associated with summer damage in Europe. Tree canopies of nectarine orchards under organic and integrated management were sampled in Lleida and Girona at four key moments of the season (bud burst, full flowering, fruit setting stage, and colorization of the fruit) during 2021 and 2022 to determine the species composition in the area and the damage caused during fruit maturation. Adult individuals in flowers, leaves, and fruit surfaces were collected and identified, and silvering damage to the fruit surface was assessed in the Lleida area. Fifteen species in Lleida and 10 species in Girona were collected from the tree canopy. Organic orchards in Lleida showed lower populations and silvering damage levels when compared with integrated orchards. *Thrips fuscipennis* Haliday (Thysanoptera: Thripidae) 1836 was the main species in Lleida during harvest, and *Frankliniella occidentalis* (Pergande) 1895 was the main species in Girona. Due to their predominance, both species were associated with silvering damage during fruit maturation.

## 1. Introduction

Nectarine is one of the main crops cultivated in Catalonia (8326 ha [1]) and Spain (28,754 ha [2]). The Lleida area constitutes up to 94% of nectarine production in Catalonia and is one of the main producer areas in Spain. Thrips are a critical nectarine pest, producing scarring, malformation, and abortion in flowering and silvering during fruit maturation. These aesthetic damages cause fruit devaluation or depreciation [3,4,5]. Damage during blossom is related to different thrips species, such as *Thrips meridionalis* (Priesner) 1926, *Thrips tabaci* Lindeman 1889, *Thrips major* Uzel 1895, *Thrips angusticeps* Uzel 1895, *Taeniothrips inconsquens* (Uzel) 1895 [3,4], *Thrips minutissimus* L. 1758 [3], *Frankliniella intonsa* Trybom 1895, and *Thrips obscuratus* (Crawford) 1941 [6,7].

Damage in nectarine gained importance in blossom and during fruit maturation with the expansion of *Frankliniella occidentalis* (Pergande) 1895 in Spain [8,9]. *F. occidentalis* is active throughout the year in coastal and warm areas of Spain, while in inland regions, as in the Ebro basin area, its proliferation stops in the winter period, to resume activity in the spring, approximately during the flowering period. Population levels peak during June and July, and their activity can continue into autumn, October or November, being present in trees or weeds, depending on the temperatures [9]. This species is considered the main pestiferous thrips in nectarine [5]. Nevertheless, in Piemont (Italy), *Thrips fuscipennis* Haliday 1836 was related to damage in fruit maturation during the early 2000s [10], and *Th. obscuratus* (Crawford) 1941 in New Zealand is responsible for both blossom and maturation damage [6]. In different parts of the world, different crops are cultivated, and different ground cover species are present, which implies that different native thrips fauna are present. As a result, the species recorded as pests also differ [11]. This fact highlights the need to determine the thrips species present in every area and crop, and those related to its damage.

Regarding the thrips species composition in nectarine orchards, *Th. obscuratus*, *Limothrips cerealium* Haliday 1836, *Haplothrips niger* (Osborn) 1883, *Thrips australis* (Bagnall) 1915, *Chirothrips manicatus* Haliday 1836, *Tenothrips frici* (=*Ceratothrips frici*) Uzel 1895, *Aeolothrips fasciatus* L. 1758, *Desmidothrips walkearae* Mound 1977, *Anaphothrips obscurus* (Müller) 1776, *F. occidentalis*, and *Thrips vulgatissimus* Haliday 1836 were found in studies of the seasonal activity of thrips in stone fruit orchards in New Zealand [7]. In the East Mediterranean region (Turkey), *F. occidentalis*, *F. intonsa*, *Th. tabaci*, *Th. major*, *Th. meridionalis*, *Th. minutissimus*, *Th. angusticeps*, *Th. australis*, *Thrips hawaianensis* (Morgan) 1913, *Melanthrips* spp. Haliday 1836, *Melanthrips fuscus* (Sulzer) 1776, *Melanthrips pallidor* Priesner 1919, *Te. frici*, *Tenothrips discolor* (Karny) 1907, *Aeolothrips intermedius* Bagnall 1934, *Mycterothrips tschirkunae* Yakhontov 1961, *Oxythrips ajugae* Uzel 1895 and, *Haplothrips aculeatus* (Fabricius) 1803 inhabit nectarine flowers [12,13]. In North Cyprus, *Aeolothrips collaris* Priesner 1919, *Aeolothrips gloriosus* Bagnall 1914, *C. manicatus*, *F. occidentallis*, *Haplothrips bolacophilus* Priesner 1938, *Haplothrips flavinctus* Karny 1910, *Haplothrips flavitibia* Williams 1916, *Isoneurothrips australis* Bagnall 1915, *Limothrips angulicornis* Jablonowski 1894, *L. cerealium*, *Me. fuscus*, *My. tschirkunae*, *Neohydatothrips gracilicornis* (Williams) 1916, *Odontothrips confusus* Priesner 1926, *Ox. ajugae*, *Te. discolor*, *Th. angusticeps*, *Th. major*, *Thrips mareoticus* (Priesner) 1932, *Th. minutissimus*, and *Th. tabaci* were cataloged [14]. In Emilia Romagna (Italy), *Th. major*, *Th. tabaci*, *F. occidentalis*, *Caliothrips fasciatus* (=*Heliothrips*) (Pergande) 1895, and other *Thrips* spp. L 1758 were found [15]. In Spain, *F. occidentalis*, *Th. tabaci*, *Th. angusticeps*, *Th. meridionalis*, *Th. major*, *Odontothrips ignobilis* Bagnall 1919, *Te. discolor* (=*C. discolor*), *Scirtothrips inermis* Priesner 1913, *Scolothrips longicornis* (Bagnall) 1909, *An. obscurus*, *C. manicatus*, *Me. fuscus*, and *Aeolothrips tenuicornis* Bagnall 1926 were collected in the flowers and branches of nectarine in orchards in Murcia [9]. Additionally, *Th. fuscipennis*, *Frankliniella tenuicornis* (Uzel) 1895, *Ae. fasciatus*, and *Ae. intermedius* are cited as sporadic inhabitants of stone fruit flowers [4].

No studies have addressed the species composition in nectarine orchards in the Lleida and Girona provinces. In the Lleida province surveyed in this study, *F. occidentalis*, *Th. tabaci*, *Th. major*, *An. obscurus*, *Sericothrips staphylinus* Haliday 1836, *Te. frici*, *Aeolothrips* spp. Haliday 1836, *Chirothrips* spp. Haliday 1836, and unidentified species of the suborder Tubulifera were mentioned [16,17]. Recently, the incidence of thrips has been increasing in the productive area of Lleida, mainly during fruit maturation, producing significant damage to the fruits. Technicians and producers stated that, in the past years in some orchards, more than 50% of fruit production was unmarketable as fresh fruit, leading to them suffering important economic losses (Bosch, personal communication). In Girona, nectarine production is very scarce, and thrips do not represent an important pest in the area.

This study aimed to determine the species composition of thrips in the tree canopies of nectarine orchards in the areas of Lleida and Girona, with a very different affectation. Additionally, this study aimed to determine the species responsible for silvering damage in fruits during maturation in nectarine orchards in Lleida.

## 2. Materials and Methods

### 2.1. Study Area

Two areas of study were considered: the intensive productive area of stone fruit in Lleida and in the Empordà area of Girona province. Both regions have small climatic differences that translate into differences in the phenology of plants and pests [18]. The agricultural area of Lleida can be subdivided into three main areas: the intensive stone fruit production area, the pip fruit-intensive production area, and the mixed production area. Orchards with different management systems were chosen to resemble the variability of the species as much as possible. Three organic (OM) orchards and three integrated pest management (IPM) orchards in the stone fruit-intensive productive area of Lleida (Soses) were surveyed in 2021 and 2022 at four different moments in the season. The distance between the orchards ranged from 0.7 to 5.0 km. In Girona province (Empordà), four IPM and two OM orchards were sampled in 2021, and four IPM and one OM orchard were sampled in 2022 at the same phenological moment as the ones in Lleida. The distance between the orchards in Girona ranged from 1.4 to 22.0 km. The orchards’ locations and sampling dates are detailed in Supplementary Material Table S1.

All the organic orchards in Lleida had ground cover (abundant resident vegetation in two of them, and sown by the farmer, but less abundant in a third one). The resident vegetation in IPM orchards was scarce for most of the season and the orchards were mainly surrounded by other stone fruit orchards. Orchards in the Girona area were homogenous, with abundant ground cover of the resident vegetation and with no more stone fruit orchards in the surroundings. The sample was taken at four stations located at the four corners of the orchard to ensure a proper representation of the orchard variability in all the samplings. The same sampling locations (10 trees divided into two different rows) were used for every sampling and in both years.

### 2.2. Bud Burst Sampling

During bud burst (BB; BBCH scale 53), 80 shots with 10 buds per shot were collected from 40 different trees in each orchard each year. Immediately after picking, the shots were placed into marked paper bags and stored in a small fridge with cold plates for transportation to the laboratory. All thrips individuals were extracted using Berlese–Tullgren funnels (Burkard Scientific, Rickmansworth, Hertfordshire, UK).

### 2.3. Full Flowering Sampling

During full flowering (FF; BBCH scale 65), 80 shots with 10 flowers per shot were collected from 40 different trees in each orchard each year and processed in the same way as the previous shots. Additionally, the trees were sampled by beating one branch in 40 trees per orchard on a white tray (BTS). Adults falling on the tray were counted and collected using a hand aspirator.

### 2.4. Fruit Setting Stage Sampling

During the fruit setting stage (FST; BBCH scale 72), five leaves per tree from 40 different trees (200 leaves per orchard) were collected each year. The trees were sampled again using BTS.

### 2.5. Sampling in the Fruit Coloring Period

During the fruit coloring period (CA; BBCH scale 85), the leaf sampling and BTS were repeated. In addition, a visual observation of two fruits per tree (80 fruits per orchard and year) was used to evaluate the fruit damage and to sample the thrips population on the fruit surface. Each fruit was classified into a damage level according to the surface affected by silvering: 0 (<1%), 1 (1–5%), 2 (6–10%), 3 (11–15%), or 4 (>16%). Fruits with categories above 1 were considered noncommercial fruit.

All the different samplings are summarized in Table 1. All adult thrips were collected and digested using lactic acid (>95%) and slide-mounted in Hoyer’s medium (Entomopraxis, Barcelona, Spain) for morphological identification. Several identification keys were used to identify the different species [4,19,20,21,22,23,24].

### 2.6. Data Analysis

Data on species correspond to all adult thrips collected using the different methodologies as a whole, not taking the methodology into consideration. The Shannon–Wiener index, Shannon uniformity, and Simpson index were calculated according to the formulas in Table 2, extracted from [25,26] to evaluate the diversity in the OM and IPM orchards in Lleida. For Girona orchards, as not enough replicates for every management and year existed, the diversity index was calculated without considering management. The different indexes were calculated using data from the whole season and field, and the means were calculated for every management. Thrips captured in the different samplings were analyzed using a generalized linear model (family Poisson).

Data from total thrips individuals (adults and immature stages) on the fruit surface were transformed to accomplish assumptions, and a Student’s *t*-test (α = 0.05) was used to compare the densities in both managements. Data on the damage to the fruit surface, expressed by the percentage of commercial fruit (damage levels 0 and 1 together), were compared between managements using a Mann–Whitney U test (α = 0.05). All the analyses were performed in R 4.2.1.

## 3. Results

### 3.1. Thrips Species Composition

#### 3.1.1. Lleida Province

Throughout the samplings, 15 species of thrips were recorded (Table 3 and Table 4). Eleven of these species were recorded in OM orchards (Table 3), while 13 were recorded in IPM orchards (Table 4). Considering the values of the Shannon–Wiener (Table 5) index, OM orchards had a higher diversity than IPM orchards. According to the Simpson index, diversity in IPM orchards was highly influenced by dominance. Shannon’s evenness showed that the uniformity was higher in OM orchards than in IPM orchards. Populations were significantly lower in 2021 than in 2022 for all the samplings (Table 6).

No thrips were found in OM orchards, and only one individual of the *Thrips* spp. was found in IPM orchards in the BB sampling in 2021. Only *Th. angusticeps* was found in OM orchards, while *Th. fuscipennis*, *Th. tabaci*, *C. manicatus*, and individuals of the *Thrips* spp. were cataloged in IPM orchards in 2022. *Th. fuscipennis* was the only species with significant population levels, having 5.67 individuals/orchard, while all the rest had 0.33 individuals/orchard.

Five species known to damage nectarine were recorded in full flowering (FF). Only *F. intonsa* was recorded in 2021, and *Th. fuscipennis*, *F. occidentalis*, *Th. angusticeps*, and *Th. meridionalis* were recorded in 2022 in OM orchards. In IPM orchards, *Th. angusticeps* was recorded in 2021, and *Th. fuscipennis* and *F. occidentalis* were recorded in 2022. Two predatory species were also cataloged: *Ae. tenuicornis* (in IPM orchards in 2021) and *Ae. intermedius* (in IPM and OM orchards in 2022). Additionally, eight thrips species not related to damage in nectarine in the literature were identified: *Te. frici* (in OM and IPM orchards in 2022); *Me. fuscus* (in OM orchards in 2022); *C. manicatus* and *Chirothrips meridionalis* Bagnall 1927 (both in IPM orchards in 2022); individuals of the *Anaphothrips* spp. Uzel 1895 (in OM and IPM orchards in 2022, individuals in IPM orchards were identified as *An. obscurus*); individuals of the *Mycterothrips* spp. Trybom 1910 (in IPM orchards in 2022); and individuals of the family Phlaeothripidae (in IPM orchards in 2021 and 2022). Populations were significantly lower in 2021 in the OM and IPM orchards than in 2022 (Table 6). Still, there were no differences in the population levels among the different managements during flowering sampling in either year. *Th. fuscipennis* was the most abundant species in both types of orchards, but in OM orchards, *F. occidentalis* and *Th. angusticeps* achieved similar levels (Table 3 and Table 4).

Four species related to damage to nectarine were recorded in the fruit setting stage (FST) sampling. In OM orchards, *Th. fuscipennis* and *Th. tabaci* were recorded in 2021, and *Th. fuscipennis*, *F. occidentalis*, and *Th. angusticeps* in 2022. In IPM orchards, *Th. fuscipennis* was recorded in 2021, and *F. occidentalis* in 2022. The only predatory thrips species was *Ae. intermedius*, recorded in 2022 in IPM orchards. Additionally, five species unrelated to nectarine damage were recorded: *Te. frici* (in 2021 only in OM orchards, and in 2022 in OM and IPM orchards); *An. obscurus* (in 2022 in both OM and IPM orchards); *C. manicatus* (in IPM orchards in 2022); individuals of the *Mycterothrips* spp. (in IPM orchards in 2021 and OM and IPM orchards in 2022, some of these individuals in IPM orchards could be identified as *Mycterothrips albidicornis* Knechtel 1923); and individuals of the family Phlaeothripidae (in OM and IPM orchards in 2022). Population levels were similar in 2021 and 2022 in both management groups (Table 6).

Four species related to damage in nectarine were recorded in the fruit coloring period (CA): *Th. fuscipennis*, *F. occidentalis*, and *Th. tabaci* were cataloged in OM and IPM orchards, while *F. intonsa* was found only in IPM orchards. *F. intonsa* was represented by only one individual. The predatory species *Ae. tenuicornis* was recorded in IPM orchards in 2021 and *Ae. intermedius* was recorded in OM orchards in 2021 and OM and IPM orchards in 2022. Additionally, six species not related to damage to nectarine were recorded: *Te. frici* (in OM and IPM orchards in both 2021 and 2022); *C. manicatus* (in IPM orchards in 2022), *Thrips albopilosus* Uzel 1895 (in IPM orchards in 2022); individuals of the *Mycterothrips* spp. (in OM and IPM orchards in 2021 and 2022, some individuals in OM orchards in 2022 were identified as *My. albidicornis*); individuals of the *Thrips* spp. (in 2021 in OM orchards); and individuals of the family Phlaeothripidae (in 2022 in OM orchards).

#### 3.1.2. Girona Province

In the Girona Empordà area, up to 10 species (Table 7) were cataloged throughout the season, considering 2021 and 2022. The diversity indexes (Table 8) showed a slightly higher diversity in 2022 than in 2021. Still, Girona province had uniformity among the populations of the different species present, with no highly dominant species.

Two species related to damage in nectarines were cataloged in the BB sampling: *F. occidentalis* and *Th. angusticeps*, although they were found only in 2022. This sampling was not possible during 2021. Additionally, individuals belonging to the family Phlaeothripidae were recorded during this sampling in 2022. The population was low for all the species (<1 individual/orchard).

Six species related to nectarine damage were recorded in the FF sampling: *F. occidentalis* (only in 2022), *Th. major* (only in 2021), *Th. tabaci* (only in 2022), *Th. fuscipennis* (only in 2022), *Th. minutissimus*, and *Th. angusticeps*. Additionally, *Me. fuscus* (only in 2021) and *Thrips* spp. were recorded. Only *Th. angusticeps* in 2021 reached levels above one individual per orchard.

Four species related to damage, namely *F. occidentalis*, *Th. major* (only in 2021), *Th. tabaci* (only in 2022), *Th. minutissimus*, and *Th. angusticeps* (only in 2022), were recorded in the FST sampling. Additionally, *Me. fuscus* (only in 2022) and individuals belonging to the family Phlaeothripidae (only in 2022) were cataloged.

Five damage-related species were recorded in the CA sampling: *F. occidentalis*, *Th. tabaci*, *Th. fuscipennis* (only in 2021), *Th. major*, and *Th. angusticeps*. Two predatory species, *Ae. intermedius* (only in 2021) and *S. longicornis* (only in 2022), were cataloged and found in 2021 and 2022. The last species was a spider mite predator [3,4]. Additionally, *Te. frici* and individuals belonging to the family Phlaeothripidae were recorded. The population levels of these individuals were scarce, as in all the seasons.

### 3.2. Damage and Population Levels at Harvest Time

OM orchards had a significantly higher proportion of commercial fruits (Table 9) than IPM orchards in both years (Mann–Whitney U test: W = 36; *p* = 0.0022). Related to this, the population on the fruit surface was significantly lower in OM orchards compared with IPM orchards (Student’s *t*-test t = 7.7317; df = 10, *p* = 1.586 × 10^−5^).

## 4. Discussion

The two-year sample revealed variations in the number of individuals recorded during the BB, FF, FST, and CA samplings between 2021 and 2022 in Lleida, with the first year having a significantly smaller population than the second. These differences might be related to some climatic differences between the years that resulted in a delay in thrips population development during the winter and spring.

According to the different diversity indexes calculated in this study, OM and Girona province orchards had a similar species richness to IPM orchards. In contrast, a high dominance in IPM orchards in Lleida translated into low diversity, as reflected in the Simpson index. Overall, the Shannon–Wiener index was always lower than the normal range for the wildlife ecosystems (1.5–3.5) [25]. Such a result is presumable as this kind of index is more suitable for stable wildlife ecosystems than highly perturbated environments like commercial orchards. Nevertheless, fruit orchards are stable year to year, and perturbation is more related to weeds, pest management, and heavy machinery movement. This nearly constant perturbation is detrimental, translating to lower diversities than expected for wildlife ecosystems.

More species were found in IPM orchards than in OM orchards in the sampled orchards in Lleida in the BB sampling. Individuals found in the BB sampling were supposed to be individuals who exited overwintering; thus, they looked for places to breed and feed. In OM overwintering, individuals might fly onto the ground cover rather than to the trees with neither flowers nor leaves. In IPM orchards where the ground cover was null by this time of the year, they might fly to the trees looking for flowers or leaves, but not all thrips species reproduce or feed there. This condition might be especially the case for *Th. Angusticeps*, an anthophilous species, and *C. manicatus*, which feeds on the flowers of Poaceae [4,27]. *Th. tabaci*, a species associated with stone fruits and flowers, and *Th. fuscipennis*, associated with Rosaceae, might exit overwintering while the trees are about to bloom. Also, *Th. fuscipennis* overwinters as adult females in dry shoots or under the scorch [3,4], its presence at this moment might indicate the species overwintering directly in *Prunus* spp. trees. The absence of *F. occidentalis* in this sampling might have resulted from the generally cold winter in Lleida, resulting in a late overwintering exit for this species. A similar pattern has been observed in the interior areas of Murcia (another autonomous community, 500 km south of the study), where *F. occidentalis* exits by the end of March and starts breeding in early April due to low winter temperatures [9].

More species appeared with tree and ground cover blossoms in the FF sampling, as expected. *Th. fuscipennis* and *F. occidentalis* were the main species associated with damage to nectarine. *F. occidentalis* individuals may belong to the overwintering generation or the first generation of the season. Only one individual of *Th. meridionalis* appeared in this sampling. This species was cited as one of the main inhabitants of stone fruit flowers [3,4], but it was rarely sampled in this study. *Me. fuscus* was mainly associated with Brassicaceae [4], and *Te. frici* was primarily associated with Asteraceae. *C. manicatus*, *C. meridionalis*, and *An. obscurus* were associated with Poaceae [4,27] and were, most probably, ground-cover inhabitants and were not breeding on nectarine.

One of the methods used to process samples from the BB and FF samplings, Berlese–Tullgren funnels, might have not been optimal for thrips. This method is used especially in the case of leaf litter samples [28] but may not be as useful in plant tissue with tight spaces where thrips can hide. The dissection of flowers and buds under a stereomicroscope would have been a more suitable methodology. Nevertheless, it has been used in flowers in other studies [9]. In addition, during the FF sampling, the collection of thrips was completed using BTS sampling.

The species found in both managements in the FST sampling were, overall, the same. *Th. fuscipennis* and *F. occidentalis* were resident in the field, although in 2022 *Th. fuscipennis* was not found in IPM orchards. FST sampling marks the start of the treatments to prevent damage in nectarine. Therefore, the populations and compositions of this species found in this sampling were expected to be highly conditioned by the treatments. The absence of *Th. fuscipennis* in 2022 IPM orchards at this time, having been present in abundance in the previous sampling, could reflect this effect. Additionally, *Th. tabaci* and *Th. angusticeps*, species related to damage in nectarine during blossoms, were present in OM orchards. All the other species were not nectarine pests but inhabitants. Individuals of the *Mycterothrips* spp. were uncommon but were present throughout the study. The individuals of *My. albidicornis* breed on the leaves of deciduous trees [20,23] and, therefore, could be breeding in nectarine trees, but with only one exception: species of this genus have not been related as pests [11]. Phlaeothripidae has a wide diversity of feeding behaviors. Some species feed on fungus hyphae, some are predators of mites and small arthropods, and some feed on the pollen and leaves of weeds [29]. Today, no species of this family have been related to nectarine damage.

The thrips population reached their maximum for *Th. fuscipennis* and *F. occidentalis* in the early summer in the CA sampling, profiting from the fruits and leaves. In this sampling, there was considerable dominance of *Th. fuscipennis* in the tree canopy. Due to the considerable difference in the capture of the two species, it is assumed that both species are responsible for the silvering damage. Still, *Th. fuscipennis* was the major cause of such damage in the study area. In this study, the immature stages of thrips on the fruit surface were not taxonomically identified, which is the most trustworthy indicator of the relationship between species and damage [11]. Nevertheless, we can assume that most of the sampled immature stages of the fruit were *Th. fuscipennis* offspring, since this species represented more than 90% of the adults sampled in the fruit.

Unlike the Lleida case, *F. occidentalis* appeared in Girona in the BB sampling. Girona province is a coastal area with mild winters. Similar differences between the coastal and interior areas have been observed in Murcia, where in the interior areas, *F. occidentalis* exits overwintering later in the season compared with coastal areas already present before flowering [9]. Despite the differences in climate between Murcia and the areas studied in the present work, it is reasonable to assume that the observed differences are due to winter conditions.

During FF sampling, most species found were related to flowering damage, but the composition differed from that in Lleida. *F. occidentalis* was already present in the orchards, indicating that it would have more importance in flowering damage than in Lleida, where they started to exit overwintering during flowering. *Th. major* and *Th. minutissimus* are typical inhabitants of stone fruit orchards [9], with *Th. minutissimus* among the first species to exit overwintering in Spanish temperate interior regions [4]. Still, none of these species were found in Lleida orchards. On the contrary, *Me. fuscus* is related to Brassicaceae, especially to *Diplotaxis erucoides* [4], which is widespread and abundant in Girona.

During FST sampling, population and species composition were similar to those found during FF sampling, although some species, such as *Th. fuscipennis* and *Te. frici*, which were present during FF sampling, were not captured during this sampling. These changes might be related mainly to ground cover changes and nectarine flower falling.

In Girona, *F. occidentalis* was the main species associated with damage in the CA sampling, although *Th. fuscipennis* also occurred in low densities. Nevertheless, the number of thrips in the orchards was lower than in the Lleida orchards. The nectarine production area in Girona was scarce compared with Lleida, where peaches and nectarines represented almost a monoculture in the sampled area. Additionally, there was dense and diverse ground cover during CA in all the Girona area sampled orchards. The possible climatic differences from Lleida may provide an advantage for the western flower thrips rather than *Th. fuscipennis*. Two predatory species were found in this sampling: *Ae. intermedius* and the spider mite predator, *S. longicornis*. In Girona, the presence of predatory thrips on nectarine was lower than in Lleida, following the overall lower densities of thrips in the tree canopy.

*Th. fuscipennis* was found to be the main species during the whole season in the intensive production area of Lleida in both OM and IPM orchards but not in Girona. Populations in Lleida were dominated by *F. occidentalis* in 2013 [16,17], but there has been a switch to populations dominated by *Th. fuscipennis*. *Th. fuscipennis* is a paleartic polyphagous species with a preference for the Rosaceae species [4,23,27]. This species has been indicated to cause damage to melon and cucumber leaves, forage legumes in flowers, strawberries, *Dianthus*, and roses (damaging the flowers, shoots, and leaves) [3,4,29]. Although little is known about the role of this species in nectarine, its presence in *Prunus* spp. in Spain is not new [4], and damage to flowers and fruits has previously been observed [3,10].

As far as we know, this is the second case of a population dominated by *Th. fuscipennis* in nectarine with associated silvering damage. The extent of this switch should be studied in further work, as we only sampled in the very specific area of stone fruit-intensive production in Lleida. Still, we do not have data from all the stone and pip fruit productive areas of the Ebro basin (Lleida and Huesca provinces). The reasons for the switch in species dominance are not known at the moment. Multiple mechanisms mediated by biotic and abiotic factors may drive displacements [30], for example, an insecticide-mediated artificial selection [31,32], interspecific competition [33], or a small change in climatic patterns at a local scale [34], among others. This displacement has had a significant impact on pest management in the area, making thrips control difficult due to the low response to the insecticide treatments strategy stated by the technicians.

Apart from *Th. fuscipennis* and *F. occidentalis*, *Th. tabaci*, a highly polyphagous and widely studied thrips species in horticultural crops, was also found on the fruit surface. This species is able to reproduce in nectarine shoots [9]. However, no bibliography has considered *Th. tabaci* to be responsible for silvering damage in nectarine, similar damage has occasionally been observed in peas associated with this species [35].

Although the flowering damage was not taken into consideration in the present study, because the economic losses in the study areas are mainly due to silvering, it has been observed that a wide range of species associated with this type of damage appeared in the samplings: *F. occidentalis*, *Th. tabaci*, *Th. fuscipennis*, *Th. meridionalis*, and *Th. angusticeps* in Lleida and *F. occidentalis*, *Th. major*, *Th. minutissimus*, *Th. tabaci*, and *Th. angusticeps* in Girona [4,8,10]. Different from silvering damage, which has, as far as we know, few species involved, flowering damage may be dependent on a wide species complex.

The predatory species, *Ae. intermedius* and *Ae. tenuicornis*, were present for most of the season, albeit in very few numbers. However, their potential involvement in the control of phytophagous thrips found in nectarine trees cannot be dismissed. *Ae. intermedius* needs to feed on floral structures to reproduce [33]. Thus, they might not find nectarine trees suitable hosts after blossoming, as pollen will not be provided to juveniles. The species of *Aeolothrips* crawl within the host looking for prey [36], different from the phytophagous species that tend to aggregate to feed and mate [37,38]. This condition may lead to individualized behavior and more frequent movement, which could explain the low number of captures. The presence in the trees of *Aeolothrips* immature stages and, therefore, if effective reproduction exists, is unknown.

Regarding the population density and the fruit damage levels in IPM and OM orchards during harvest, the lower population density in OM orchards and, consequently, the low damage, could be related to differences in management between the two systems. Insecticides might be one of the main differences between the two managements. Insecticide sprayings have been the basis of thrips management in many crops [37] and in IPM nectarine orchards. The efficacy at a population level might not be as good as expected due to thigmotactic behavior (i.e., hiding between touching fruits, inside flowers, or within the fruit petiole), the rapid increase in populations, and insecticide resistance [39], but natural enemies can be affected by sprayings compromising natural biological control and therefore leading to the rise in pest levels [40,41,42]. Additionally, OM orchards generally had denser ground cover and lower thrips populations than integrated orchards. Therefore, ground cover could have an effect, either through hosting thrips or as a reservoir for natural enemies. *F. occidentalis* strongly prefers ground-level flower blooms rather than medium or high ones. Although no important effect of ground cover on the populations of nectarine trees has been demonstrated, the joint effect with other factors is still unknown [43]. It has been proven that the sowing of flowering plants in fruit orchards can have an effect on beneficial species populations, including thrips predators such as *Anthocoris nemoralis* Fabricius 1794 (Hemiptera: Anthocoridae) or *Orius* spp. [44,45,46]. In greenhouse trials, the use of *Calendula officinalis*, together with the release of *Orius sauteri* (Poppius 1909) (Hemiptera: Anthocoridae), improved the control of *F. occidentalis* in tomato [46]. Looking at both the hypotheses simultaneously, low chemical pressure and dense ground cover may result in improved control of the thrips population in nectarine trees by improving natural biological control or by providing more attractive hosts for thrips.

From a global view, the species diversity in nectarine orchards seems homogeneous within the Mediterranean region. The species found in this study are similar to the species or genera that other studies have found [9,12,13,14,15]. The damaging and spontaneous species associated with the resident vegetation were identical.

Species not feeding or breeding in nectarine but in weeds are usually found in trees as an accidental species. This is the case for *An. obscurus*, *C. manicatus*, *C. meridionalis*, *Me. fuscus*, *Te. frici*, and Phlaeothripidae species, which were individually found in this study and in others [7,9,14,16,17]. As observed in [47], we observed species associated with Poaceae that are not expected to develop in the canopy of nectarine trees, which may be used for generalist predatory mites and generalist predators, such as *Orius* spp., as alternative prey. This is the idea behind [48], which studied the possible use of *Festuca arundinacea* (Poaceae) to increase the *An. obscurus* population as an alternative prey for generalist predatory mites to better control *Tetranychus urticae* Koch 1836 populations in clementine orchards. The results showed that *F. arundinacea* covers could increase the *An. obscurus* and phytoseiidae populations.

The presence of weeds in the orchard at harvest time has been widely discouraged [49], but the destruction of weeds can create a problem in many cases by forcing thrips dispersal to crops [50]. Therefore, the ground cover effect on thrips diversity and population may be worthy of study as it may offer opportunities to develop a more sustainable strategy for thrips management.

We did not intend to study the temporal and spatial patterns of thrips species in this study. Therefore, no conclusions can be made on this topic. If an IPM program is expected to be developed, future research should consider studying the population dynamics of the different thrips species during the entire season. It should also be considered that thrips and natural enemies fluctuate with a strong relationship with the flowering weed species and crops surrounding orchards [44,51], which could be important at a local scale and could be useful in developing a control program based on biological control.

## 5. Conclusions

*Th. fuscipennis* was the dominant species and the major species responsible for silvering damage to nectarine in intensive productive areas of Lleida and can be considered a new nectarine pest. *F. occidentalis* occupied this niche in Girona, where *Th. fuscipennis* appeared as an occurring species. In Lleida, this species displacement is making the actual pest management difficult, requiring a rethinking of the management strategy. Even though within the Mediterranean region, the species composition seems to be in accordance with that found in the present study, the role of ground cover should be studied. Thus, understanding its interaction with nectarine trees could be useful in developing an IPM program for thrips control. The number of thrips in the canopy of the nectarines and, therefore, the fruit damage in OM orchards, was significantly lower than in IPM orchards. However, the reasons for this difference remain unclear. This is the first study on the species composition of thrips in nectarine in the Lleida and Girona provinces.

## Figures and Tables

**Table 1:** Summary of the different samplings of nectarine trees (BB = bud burst, FF = full flowering, FST = fruit setting stage, CA = fruit coloring). X = sample collected in each sampling.

	BB	FF	FST	CA
**Branches**	X	X		
**Leaves**			X	X
**Fruits**				X
**Beating**		X	X	X

**Table 2:** The diversity indexes considered to study the thrips species composition in nectarine orchard trees in the provinces of Lleida and Girona (Spain). pi = probability of finding species i, ni = number of captures of species i, N = the total number of captures, Nmax = the number of captures of the most abundant species.

Index	Formula	Measures	Interpretation
Species Richness	S = number of species	Richness	High values indicate high diversity
Shannon–Wiener (H’)	−Σp_i_lnp_i_	Richness	High values indicate high diversity
Shannon Uniformity (E)	H’/lnS	Uniformity	Values close to 1 indicate high diversity
Simpson (D)	1 − Σ(n_i_(n_i_ − 1)/N(N − 1)	Dominance	Values close to 1 indicate high diversity and low dominance

**Table 3:** Thrips species in organic nectarine orchards in Lleida province (Spain) in the different samplings during 2021 and 2022 (mean individuals/orchard ± SE). BB = bud burst, FF = full flowering, FST= fruit setting stage, CA = fruit coloring.

Family	Thrips Species	2021				2022			
		BB	FF	FST	CA	BB	FF	FST	CA
Aeolothripidae	*Aeolothrips intermedius*	-	-	-	0.33 ± 0.33	-	0.67 ± 0.67	-	0.33 ± 0.33
Melanthripidae	*Melanthrips fuscus*	-	-	-	-	-	0.33 ± 0.33	-	-
Thripidae	*Anaphothrips* spp.	-	-	-	-	-	0.33 ± 0.33	-	-
	*Anaphothrips obscurus*	-	-	-	-	-	-	0.33 ± 0.33	-
	*Frankliniella intonsa*	-	0.33 ± 0.33	-	-	-	-	-	-
	*Frankliniella occidentalis*	-	-	-	1.67 ± 0.88	-	2.33 ± 0.33	1.00 ± 1.00	0.67 ± 0.33
	*Mycterothrips* spp.	-	-	0.33 ± 0.33	0.33 ± 0.33	-	-	0.33 ± 0.33	0.00 ± 0.00
	*Mycterothrips albidicornis*	-	-	-	-	-	-	-	1.00 ± 1.00
	*Tenothrips frici*	-	-	-	2.33 ± 2.33	-	1.00 ± 1.00	0.33 ± 0.33	1.33 ± 0.33
	*Thrips* spp.	-	-	-	0.67 ± 0.67	-	-	-	-
	*Thrips angusticeps*	-	-	-	-	0.33 ± 0.33	3.00 ± 0.58	2.00 ± 1.15	-
	*Thrips fuscipennis*	-	-	1.33 ± 0.88	35.00 ± 26.31	-	4.33 ± 2.40	3.00 ± 2.08	39.00 ± 35.50
	*Thrips meridionalis*	-	-	-	-	-	0.33 ± 0.33	-	-
	*Thrips tabaci*	-	-	0.33 ± 0.33	4.33 ± 2.03	-	-	-	1.33 ± 0.67
Phlaeothripidae		-	-	-	-	-	-	0.33 ± 0.33	0.67 ± 0.67
Not identified		-	-	-	1.33 ± 1.33	-	-	-	0.33 ± 0.33

**Table 4:** Thrips species in IPM nectarine orchards in Lleida province (Spain) in the different samplings during 2021 and 2022 (mean individuals/orchard ± SE). BB = bud burst, FF = full flowering, FST = fruit setting stage, CA = fruit coloring.

Family	Thrips Species	2021				2022			
		BB	FF	FST	CA	BB	FF	FST	CA
Aeolothripidae	*Aeolothrips intermedius*	-	-	-	-	-	0.33 ± 0.33	1.00 ± 0.58	0.33 ± 0.33
	*Aeolothrips tenuicornis*	-	0.67 ± 0.33	-	1.00 ± 1.00	-	-	-	-
Thripidae	*Anaphothrips obscurus*	-	-	-	-	-	1.00 ± 1.00	0.67 ± 0.33	-
	*Chirothrips manicatus*	-	-	-	-	0.33 ± 0.33	0.67 ± 0.33	0.67 ± 0.33	0.33 ± 0.33
	*Chirothrips meridionalis*	-	-	-	-	-	0.33 ± 0.33	-	-
	*Frankliniella intonsa*	-	-	-	0.33 ± 0.33	-	-	-	-
	*Frankliniella occidentalis*	-	-	-	7.67 ± 6.69	-	1.67 ± 0.67	1.33 ± 0.88	16.33 ± 3.84
	*Mycterothrips* spp.	-	-	0.33 ± 0.33	0.33 ± 0.33	-	0.33 ± 0.33	2.33 ± 1.33	0.67 ± 0.67
	*Mycterothrips albidicornis*	-	-	0.33 ± 0.33	-	-	-	0.33 ± 0.33	-
	*Tenothrips frici*	-	-	-	0.33 ± 0.33	-	4.33 ± 2.40	0.33 ± 0.33	0.67 ± 0.67
	*Thrips* spp.	0.67 ± 0.67	-	-	2.00 ± 1.53	0.33 ± 0.33	-	-	-
	*Thrips angusticeps*	-	1.67 ± 1.20	-	-	-	-	-	-
	*Thrips albopilosus*	-	-	-	-	-	-	-	0.33 ± 0.33
	*Thrips fuscipennis*	-	-	2.00 ± 2.00	239.67 ± 10.17	5.67 ± 4.70	5.33 ± 4.37	-	350.33 ± 58.62
	*Thrips tabaci*	-	-	-	1.00 ± 0.58	0.33 ± 0.33	-	-	1.00 ± 0.58
Phlaeothripidae		-	0.33 ± 0.33	-	-	-	0.33 ± 0.33	0.67 ± 0.67	-
Not identified		-	-	-	2.00 ± 0.00	-	0.33 ± 0.33	0.33 ± 0.33	-

**Table 5:** Thrips species diversity index per year and management system (mean ± SE) in Lleida province nectarine orchards. OM = organic management, IPM = integrated pest management.

	2021		2022	
	IPM	OM	IPM	OM
**Species richness**	5.67 ± 1.20	4.33 ± 0.33	9.33 ± 0.67	7.67 ± 0.88
**Shannon–Wiener index**	0.27 ± 0.10	0.91 ± 0.32	0.45 ± 0.04	1.38 ± 0.44
**Shannon evenness**	0.16 ± 0.06	0.61 ± 0.22	0.21 ± 0.03	0.68 ± 0.21
**Simpson index**	0.11 ± 0.05	0.50 ± 0.20	0.18 ± 0.02	0.62 ± 0.21

**Table 6:** The number of individuals belonging to Thysanoptera captured in every sampling (mean ± SE) in nectarine orchards in Lleida province. BB = bud burst, FF = full flowering, FST = fruit setting stage, CA = coloring of the fruit. Different letters indicate significant differences within the samplings (Tukey test *p* < 0.05).

Year	Management	Captures (mean ± SE)			
		BB	FF	FST	CA
**2021**	**OM**	0.00 ± 0.00c	0.33 ± 0.33b	2.00 ± 0.58bc	44.33 ± 23.79d
**2021**	**IPM**	0.67 ± 0.67b	2.00 ± 1.15b	2.67 ± 2.67c	251.33 ± 5.93b
**2022**	**OM**	0.33 ± 0.33b	11.67 ± 4.06a	7.33 ± 3.84a	44.00 ± 34.56c
**2022**	**IPM**	6.67 ± 5.24a	14.00 ± 7.02a	6.33 ± 2.19ab	369.67 ± 62.99a

**Table 7:** Thrips species found in Girona nectarine orchards during the samplings in 2021 and 2022 (mean individuals/orchard ± SE). BB = bud burst, FF = full flowering, FST= fruit setting stage, CA = fruit coloring.

Family	Thrips Species	2021				2022			
		BB	FF	FST	CA	BB	FF	FST	CA
Aeolothripidae	*Aeolothrips intermedius*	-	-	-	0.29 ± 0.18	-	-	-	-
Melanthripidae	*Melanthrips fuscus*	-	0.71 ± 0.36	-	-	-	-	0.20 ± 0.20	-
Thrpidae	*Frankliniella occidentalis*	-	-	0.14 ± 0.14	2.71 ± 1.04	0.20 ± 0.20	0.60 ± 0.40	0.40 ± 0.24	0.80 ± 0.49
	*Scolothrips longicornis*	-	-	-	-	-	-	-	0.20 ± 0.20
	*Tenothrips frici*	-	-	-	0.86 ± 0.46	-	0.20 ± 0.20	-	0.20 ± 0.20
	*Thrips* spp.	-	0.14 ± 0.14	-	-	-	-	-	-
	*Thrips angusticeps*	-	1.14 ± 0.99	-	0.14 ± 0.14	0.20 ± 0.20	0.20 ± 0.20	0.20 ± 0.20	0.20 ± 0.20
	*Thrips fuscipennis*	-	-	-	0.14 ± 0.14	-	0.20 ± 0.20	-	-
	*Thrips major*	-	0.29 ± 0.29	0.29 ± 0.29	0.86 ± 0.70	-	-	-	0.40 ± 0.24
	*Thrips minutissimus*	-	0.14 ± 0.14	0.14 ± 0.14	-	-	0.20 ± 0.20	0.20 ± 0.20	-
	*Thrips tabaci*	-	-	-	1.71 ± 1.13	-	0.20 ± 0.20	0.20 ± 0.20	1.00 ± 0.63
Phlaeothripidae		-	-	-	0.14 ± 0.14	0.20 ± 0.20	0.40 ± 0.40	0.20 ± 0.20	0.40 ± 0.24

**Table 8:** Thrips species diversity index (mean ± SE) in Girona province nectarine orchards.

	2021	2022
**Species richness**	3.29 ± 1.02	3.80 ± 0.86
**Shannon–Wiener index**	0.88 ± 0.26	1.10 ± 0.29
**Shannon evenness**	0.19 ± 0.05	0.25 ± 0.06
**Simpson index**	0.49 ± 0.14	0.68 ± 0.17

**Table 9:** Commercial fruits (mean% ± SE) and the number of adults, immature stages, and total thrips population (mean thrips/orchard ± SE) on the fruit surface in Lleida province nectarine orchards. Different letters in the same column indicate significant differences (*p* < 0.05).

Year	Management	Commercial Fruits (%)	Adults (ind/Fruit)	Immature Stages (ind/Fruit)	All Stages (ind/Fruit)
**2021**	**OM**	96.25 ± 2.6a	0.42 ± 0.13	0.21 ± 0.19	0.63 ± 0.27b
	**IPM**	52.08 ± 17.4b	2.34 ± 1.29	1.70 ± 0.86	4.04 ± 0.55a
**2022**	**OM**	99.16 ± 0.41a	0.03 ± 0.03	0.24 ± 0.00	0.27 ± 0.03b
	**IPM**	45.72 ± 13.69b	1.10 ± 0.52	2.38 ± 0.57	3.48 ± 0.37a

## Data Availability

The raw data supporting the conclusions of this article will be made available by the authors on request.

## Supplementary Materials

The following supporting information can be downloaded at: https://www.mdpi.com/article/10.3390/insects15090699/s1. Table S1 containing the different orchards sampled, location, area, and sampling dates.
